# Low-Intensity Pulsed Ultrasound in Patients With Atherosclerotic Peripheral Arterial Disease: A Pilot Study and Randomized Double-Blind Placebo-Controlled Trial

**DOI:** 10.7759/cureus.84125

**Published:** 2025-05-14

**Authors:** Farina Mohamad Yusoff, Masato Kajikawa, Takayuki Yamaji, Shinji Kishimoto, Tatsuya Maruhashi, Ayumu Nakashima, Yukihito Higashi

**Affiliations:** 1 Research Center, Hiroshima University, Hiroshima, JPN; 2 Nephrology, Graduate School of Medicine, University of Yamanashi, Chuo City, JPN

**Keywords:** atherosclerotic, cardiovascular regenerative medicine, : lipus- low intensity pulsed ultrasound, peripheral arterial disease (pad), research in angiogenesis

## Abstract

Introduction

Low-intensity pulsed ultrasound (LIPUS) influences myogenesis, osteogenesis, and angiogenesis. The purpose of this study was to evaluate the effect of LIPUS on symptoms in patients with atherosclerotic peripheral arterial disease (PAD).

Methods

This pilot study was a double-blinded, placebo-controlled, and randomized study for investigation of LIPUS treatment in patients with atherosclerotic PAD. Thirteen subjects received active LIPUS devices, and another 13 control subjects received inactive LIPUS devices for a 24-week period. The outcomes of symptoms, along with visual analog score (VAS), ankle-brachial index (ABI), transcutaneous oxygen pressure (TcPO_2_), and skin perfusion pressure (SPP), were evaluated. Investigations of the number and migration of circulating progenitor cells (CPCs) were also performed.

Results

There was significant decrease in rest pain intensity on VAS after 24-week LIPUS treatment. During the 24-week period, LIPUS treatment had a tendency to increase ABI and TcPO_2_. There was no significant increase in SPP. No significant changes in ABI, TcPO_2_, SPP, and VAS in the control group was found. The number of and migration of CPCs were increased in patients with atherosclerotic PAD after LIPUS treatment. No severe adverse effects were observed in any of the patients who underwent LIPUS treatment.

Conclusions

LIPUS is a safe and non-invasive treatment, and it improves symptoms in patients with atherosclerotic PAD. Through ultrasound irradiation, inferences in therapeutic cellular regeneration, patients with chronic limb ischemia, including non-atherosclerotic PAD, of different severity levels can benefit from LIPUS.

## Introduction

Chronic limb-threatening ischemia (CLTI), also known as critical limb ischemia, is an advanced and complex progression of peripheral arterial disease (PAD) [1-3]. The presence of PAD is associated with limitations of physical function and walking performance and impaired quality of life. Patients with CLTI suffer from discomfort and pain over the affected limb and have poor healing of wounds or ulcers due to unceasing poor vascularity, and they are at risk of limb amputation. Approximately one-third of PAD patients with CLTI are deemed to be not suitable for conventional revascularization treatment due to anatomical limitations and comorbidities [4,5]. Therapeutic angiogenesis options, such as cell therapy and gene therapy, have been explored as treatment strategies for CLTI [6-10]. Hereto, these therapies are invasive, and there are ongoing issues regarding expertise and safety. Therefore, another innovative therapeutic option that is noninvasive and safe is needed to improve symptoms in PAD patients.

Low-intensity pulsed ultrasound (LIPUS) has been shown to influence angiogenesis, myogenesis, osteogenesis, and wound healing [10-14]. LIPUS has been applied for the treatment of bone fractures and soft tissue injuries in clinical practice. In vivo and in vitro experimental studies have shown that LIPUS enhances angiogenesis through activation of the extracellular signal-regulated kinase (ERK)/Akt/endothelial nitric oxide synthase (eNOS)/vascular endothelial growth factor (VEGF) signaling pathway [10]. LIPUS irradiation may have beneficial effects on symptoms of limb ischemia in patients with PAD through its influence on angiogenesis [14]. In the current study, the effects of LIPUS treatment on symptoms in patients with atherosclerotic PAD were evaluated.

## Materials and methods

Study population

**Figure 1:**
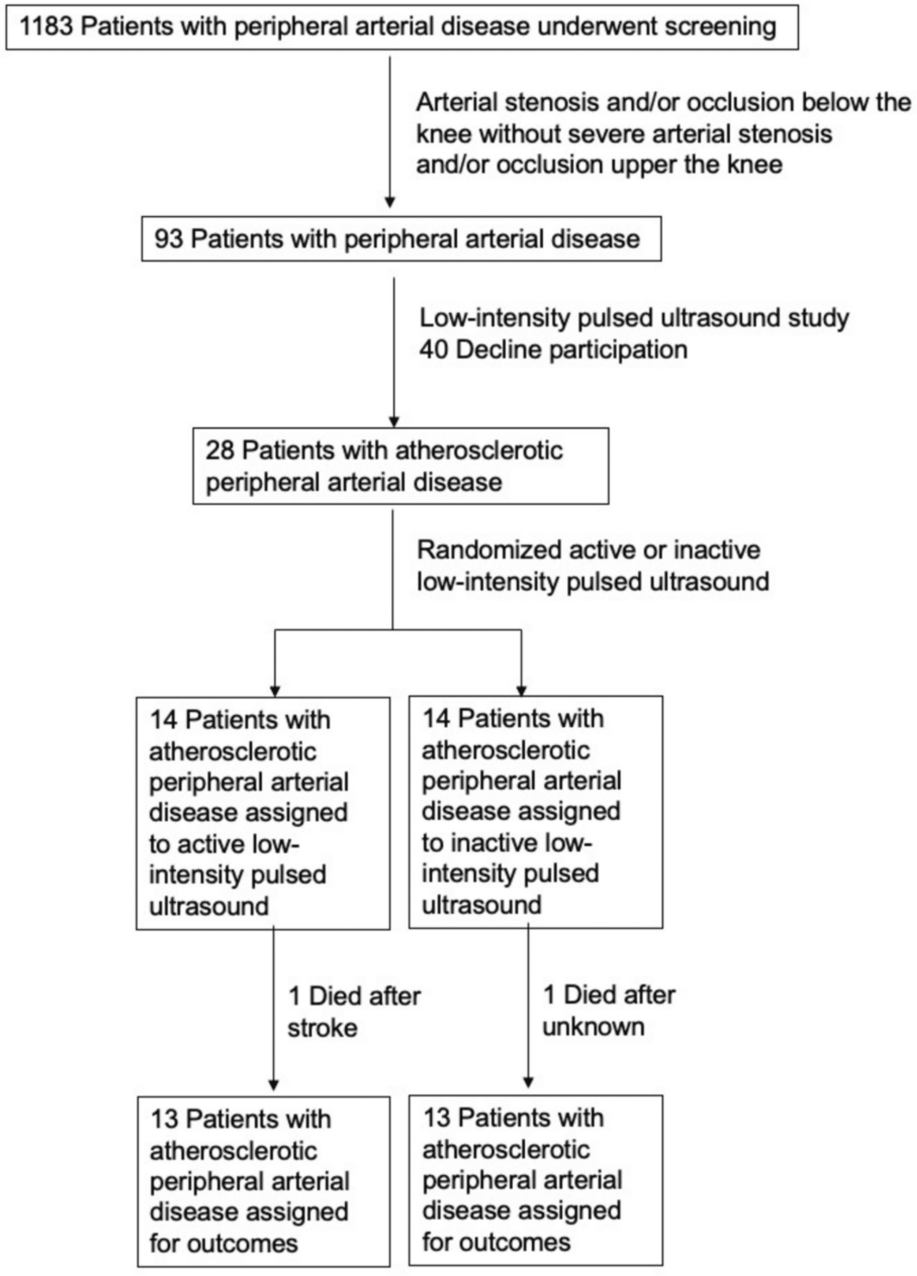
Flow chart of the study design from screening to completion of the trial.

Twenty-eight patients with atherosclerotic PAD who had arterial stenosis and/or occlusion below the knee from the Hiroshima University PAD database were enrolled in this study (Figure 1). The inclusion criteria were patients with severe ischemic peripheral vascular disease. The exclusion criteria were patients with a recent history of malignant disease within five years prior to the study, patients with stroke and/or myocardial infarction within 12 weeks prior to the study, patients with heart failure (NYHA4), and other vasculitis and hypercoagulable states. Evaluation of the rheumatoid factor, lupus anticoagulants, serologic investigations, and angiography was performed. Patients with arterial stenosis and/or occlusion below the knee without severe arterial stenosis and/or occlusion upper the knee were included in the recruitment process. Among these patients, 40 potential subjects declined participation due to various issues, including inability to participate in study schedules.

Study protocol

This pilot study on patients with atherosclerotic PAD was a double-blinded and randomized study with active and inactive LIPUS. As previously described in Mohamad Yusoff et al. [14], a LIPUS device with an individual transducer (Nippon Sigmax Co., Ltd., Tokyo, Japan) generating a pulsed 2.0-MHz signal with a 200-μ second pulse burst and 1-kHz repetition rate at the intensity of 30 mW/cm^2^ and with a duty cycle of 20% of pulse wave (Supplementary Figure S1: Figure 5). All subjects were taught how to properly operate the device. The ultrasound irradiation was applied over the skin at the calf region of the ischemic limb at home, 20 minutes every day for 24 weeks. The gap between the transducer and skin was filled with ultrasonic gel [14]. Eight transducers were attached to the skin over the gastrocnemius of each ischemic leg. Dummy LIPUS devices were prepared as inactive LIPUS. The inactive LIPUS device had the same appearance as the LIPUS device, with a dummy indicator light that turned on in operation. Twenty-eight patients with atherosclerotic PAD were randomly divided into an active LIPUS group (14 patients with atherosclerotic PAD) and an inactive LIPUS group (14 patients with atherosclerotic PAD). The study protocol was approved by the Ethics Committee of Hiroshima University, and the registration number for the clinical trial was UMIN000004901. Written informed consent for participation in the study was obtained from all subjects.

Randomization and masking 

Eligible patients were randomly assigned to receive treatment with either active LIPUS equipment (Nippon Sigmax Co., Ltd., Tokyo, Japan) or dummy LIPUS equipment (ratio 1:1), as previously described [14]. The random code list was electronically generated by an independent trial statistician with the clinical center being assigned as a block. Randomization was managed by a third-party enrollment center.

Digital subtraction angiography (DSA) 

All subjects underwent intra-arterial DSA for examination of PAD. Briefly, DSA was performed with a 4-F pigtail catheter (TERUMO, Tokyo, Japan) by using DSA units (Siemens-Asahi Medical, Tokyo, Japan). With the tip of the catheter placed at the common iliac arteries, images include the thighs, knees, and crural regions were captured. At each station, 20 mL of iopromidum was injected at a rate of 10 mL/sec. The criteria of limb ischemia by angiography include disrupted and reduced blood flow with abrupt occlusion, localized stenosis, bridging collaterals, and run-off at the peripheral side of the lesion in the affected limb.

Measurements of endpoints 

The primary endpoint was the change in rest pain intensity on a visual analog scale (VAS) from baseline up to 24 weeks. The secondary endpoints were changes in assessment of VAS from baseline up to 12 weeks, changes in transcutaneous oxygen pressure (TcPO_2_), skin perfusion pressure (SPP), ankle-brachial index (ABI), and examinations of the number of and function of circulating progenitor cells (CPCs) from baseline up to 12 and 24 weeks, and changes in symptoms in the study period of 24 weeks. Data collection timing was at baseline (0 week of treatment), at 4, 12, and 24 weeks in both control and treatment groups. Standard clinic devices were used and were calibrated in accordance with standard practice.

Visual analog scale (VAS)

Rest pain intensity was assessed by a self-administered VAS from baseline to 24 weeks, as previously described [14,15]. In brief, the observers were asked to place a marker on the VAS line at a point that represents the pain intensity of respondents. The respondents completed the VAS line presentation by themselves. The VAS score was a horizontal line of 10 cm in length, with 0 cm corresponding to no pain and 10 cm corresponding to the most severe pain. The observers were blinded to the manner of examinations.

Perfusion parameters, TcPO_2_, SPP, and ABI

Utilizing standard examination techniques, as previously described, assessments of perfusion parameters, along with TcPO_2_, SPP, and ABI, were performed [14]. Examination of each of the subjects was performed in a supine position in a dimly lit, air-conditioned, and quiet room in the morning after overnight fasting (constant temperature of 22-25°C). Perfusion parameters were measured after a 15-minute rest period. TcPO_2_ was measured by an electrochemical transducer (TCM 400, Radiometer K.K., Tokyo, Japan), as previously described [16]. The skin at the site of measurement was shaved and cleaned with alcohol to ensure optimal TcPO_2_ electrode contact for skin oxygen diffusion. Then, the TcPO_2_ electrodes that were stabilized at 43°C were placed on the skin at the site of measurement. The TcPO_2_ values were recorded after a 20-minute stabilization period for the electrodes. SPP was measured by a SensiLase PAD3000 flowmeter (Kaneka Medix Co., Osaka, Japan), as previously described [17]. A laser Doppler probe was positioned within the blood pressure cuff wrapped around the subject’s foot. ABI was measured by a device (Form PWV/ABI; Omron Colin, Co., Tokyo, Japan). The subjects were kept in the supine position for at least five minutes, and cuffs were wrapped around both their brachia and ankles. An oscillometric method was used to measure systolic blood pressure in the bilateral brachial and posterior tibial arteries. The ABI value was calculated by dividing the highest pressure in the posterior tibial arteries on the right and left sides by the highest brachial pressure on either side. The observers were blinded to the manner of examinations.

Number of CPCs 

The number of CPCs was analyzed by flow cytometry, as previously described [18]. In brief, venous blood of subjects was collected in tubes containing sodium EDTA (7 mg/mL) and in polystyrene tubes and was chilled promptly in an ice bath. The peripheral blood mononuclear cells were then isolated by Ficoll density gradient centrifugation (AXIS-SHIELD, Dundee, Scotland). 10^6^ peripheral blood mononuclear cells were incubated for 10 minutes with monoclonal antibodies against human fluorescein isothiocyanate (FITC)-conjugated anti-CD45 (Miltenyi Biotec, Bergisch Gladbach, Germany), phycoerythrin (PE)-conjugated anti-AC133 (Miltenyi Biotec), and allophycocyanin (APC)-conjugated anti-CD34 monoclonal antibody (Becton Dickinson Biosciences, Franklin Lakes, NJ). Isotype controls and IgG subclass of each antibody were used to assess the background. After incubation and lysis of erythrocytes, the remaining cells were washed with phosphate-buffered saline and then fixed in 2% paraformaldehyde for analysis using a FACSCalibur flow cytometer (Becton Dickinson Biosciences), with each analysis consisting of 500,000 events. The mononuclear cell fraction was gated and analyzed for the expression of AC133 and CD45. Only AC133^+^CD45^low^ cells were finally investigated for the count of CD34^+^ cells.

Progenitor cell characterization

As previously mentioned, the mononuclear cells were isolated from 50 mL of peripheral blood obtained from subjects and 10^4^ mononuclear cells were plated on 6-well culture dishes coated with human fibronectin and gelatin and maintained in endothelial cell basal medium-2 (EBM-2, Cell Systems) supplemented with endothelial cell growth medium (EGM-2) microvascular single aliquots and 5% fetal bovine serum. Non-adherent cells were removed after day three of culture, and cytochemical analysis of adherent cells was performed on day four. Cultivated cells were incubated with 1,1’-dioctadecyl-3,3,3,3-tetramethylindocarbocyanine-labeled acetylated low-density lipoprotein (LDL) (Di-AcLDL; Molecular Probes, Carlsbad, CA) (10 μg/mL) at 37°C for one hour. Cells were then fixed with 2% paraformaldehyde for 10 minutes and incubated with FITC-labeled Ulex europaeus agglutinin I (lectin, 10 μg/mL; Sigma-Aldrich, St. Louis, MI) for one hour. Progenitor cells were identified by display of double-positive staining for lectin and Di-AcLDL (Supplemental Figure S2: Figure 6).

Migration assay

Isolated progenitor cells for a modified Boyden chamber assay were detached mechanically by using a cell scraper and harvested after centrifugation [18]. Resuspension was performed in 300 μL of EBM. Additionally, 2 × 10^4^ progenitor cells were placed in the upper chamber of a modified Boyden chamber (FluroBlock, Becton Dickinson Biosciences). The chamber was placed in a 24-well culture dish containing EBM and culture medium for control and human recombinant VEGF (50 ng/mL; Sigma-Aldrich). After 24 hours of incubation at 37°C, the lower side of the filter was washed with phosphate-buffered saline (PBS) and fixed with 2% paraformaldehyde. Cell nuclei were stained with 4′,6-diamidino-2-phenylindole dihydrochloride (DAPI, Sigma-Aldrich), and the number of cells that migrated to the lower chamber was counted manually in three random high-power fields. Each experiment was performed in triplicate.

Statistical analysis

Comparison between groups was performed using a t-test for continuous variables, and Fisher's exact test was applied to evaluate the relationship between categorical variables. Analysis of variance (ANOVA) was employed to compare means across groups when appropriate, followed by post-hoc tests to identify specific group differences if significant results were detected. All analyses were conducted with a significance level set at α = 0.05, and assumptions for each test were verified prior to implementation. Data were processed using JMP Pro. Ver 18 software (SAS Institute, Cary, NC).

## Results

Clinical characteristics

Baseline clinical characteristics of patients in the LIPUS group and patients in the no-LIPUS group (control group) are summarized in Table 1.

**Table 1:** Clinical characteristics of patients in the control and the low-intensity pulsed (LIPUS) groups. Data are expressed as mean ± standard deviation or number and percentage, with T indicating t-statistic and P as p-value. * indicates that Fisher’s exact test was used. Abbreviations: CRF, chronic renal failure; ACE, angiotensin-converting enzyme; ARB, angiotensin receptor blocker

Variables	Control group (n=13)		LIPUS group (n=13)		T	P
Age, year	69.9 ± 5.4		70.9 ± 4.8		0.50	0.62
Gender, men/women	12/1		12/1		*	1.00
Body mass index, kg/m^2^	21.9 ± 3.0		21.7 ± 2.7		0.18	0.85
Rutherford category, n (%)						
3	3 (23)		3 (23)		*	1.00
4	3 (23)		4 (31)		*	>0.99
5	4 (31)		3 (23)		*	>0.99
6	3 (23)		3 (23)		*	1.00
Complications, n (%)						
Hypertension	8 (62)		9 (69)		*	>0.99
Diabetes mellitus	7 (54)		7 (54)		*	1.00
Dyslipidemia	4 (13)		4 (31)		*	1.00
CRF (hemodialysis)	3 (23)		2 (15)		*	>0.99
Previous myocardial infarction	4 (31)		4 (31)		*	1.00
Previous stroke	2 (15)		3 (23)		*	>0.99
Medication, n (%)						
Anti-platelet	12 (92)		11 (85)		*	>0.99
ACE inhibitors	1 (8)		2 (15)		*	>0.99
ARBs	7 (54)		8 (62)		*	>0.99
Calcium channel blockers	4 (31)		3 (23)		*	>0.99
Statins	3 (23)		3 (23)		*	1.00
Sulfonylurea and/or metformin	2 (15)		1 (8)		*	>0.99
Insulin	3 (23)		4 (31)		*	>0.99
Smoker, n (%)	5 (38)		4 (31)		*	>0.99

ABI, TcPO_2_, SPP, and VAS 

**Figure 2:**
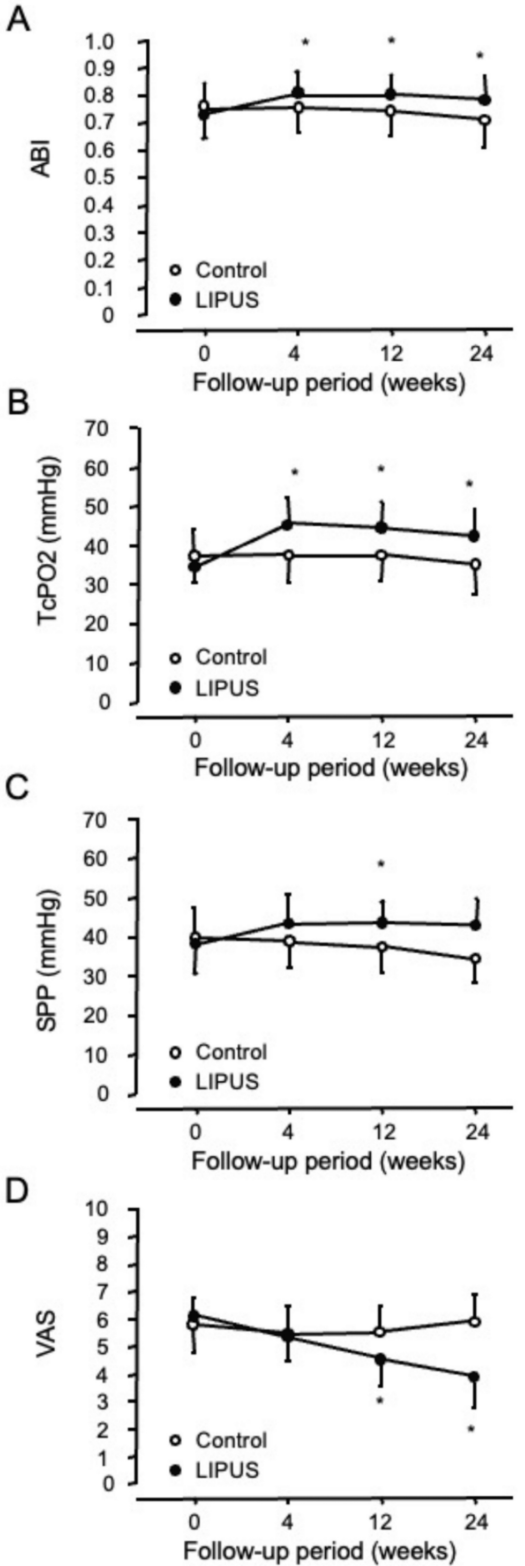
Changes in the ankle brachial pressure index (ABI), transcutaneous oxygen pressure (TcPO2), skin perfusion pressure (SPP), and visual analog pain score (VAS) in the control group (open circles) and low-intensity pulsed ultrasound (LIPUS) group (closed circles) of patients with atherosclerotic peripheral arterial disease. *p<0.05 vs. control in the same follow-up period.

Table 2 and Figure 2 show the changes in ABI, TcPO_2_, SPP, and VAS during the 24-week period of LIPUS treatment. LIPUS treatment had a tendency to increase ABI and significantly in TcPO_2_. SPP did not alter during the 24-week period in patients with PAD. The VAS was significantly decreased from 6.0±0.4 to 4.6±0.6 (p=0.01) after 12 weeks and remained significantly improved until the 24-week period of LIPUS treatment. There was no significant difference between ABI, TcPO_2_, SPP, and VAS at baseline (0 week of treatment) compared to at 4, 12, and 24 weeks in the control group. Two of the 13 atherosclerotic PAD patients treated with LIPUS had ulcers before entry into this study. The ulcers were encrusted (Supplemental Figure S3: Figure 7) after 24 weeks of LIPUS treatment.

**Table 2:** Changes in parameters in the control and the low-intensity pulsed (LIPUS) groups. Data are expressed as mean ± standard deviation, with F indicating the f-value, P as p-value, and T as t-statistic.

Variables, Groups and Tests	Before	4 weeks	12 weeks	24 weeks	F	P
Ankle brachial index, ABI						
Control	0.74 ± 0.07	0.73 ± 0.07	0.72 ± 0.08	0.70 ± 0.09	0.62	0.60
LIPUS	0.72 ± 0.08	0.80 ± 0.08	0.79 ± 0.08	0.78 ± 0.09	2.46	0.07
T	-0.68	2.37	2.23	2.27		
P	0.50	0.03	0.03	0.03		
Transcutaneous oxygen pressure, TcPO_2_ (mm Hg)						
Control	36.7 ± 7.0	36.6 ± 7.2	36.8 ± 7.1	35.2 ± 8.1	0.14	0.94
LIPUS	34.6±3.8	45.1±7.9	44.6±6.8	42.2 ± 7.2	7.00	<0.01
T	-0.95	2.87	2.86	2.33		
P	0.35	<0.01	<0.01	0.03		
Skin perfusion pressure, SPP (mm Hg)						
Control	39.1 ± 8.1	38.9 ± 7.2	37.5 ± 7.6	34.8 ± 6.5	0.94	0.43
LIPUS	38.1 ± 8.5	44.2 ± 7.9	44.3 ± 6.0	40.3 ± 7.6	2.12	0.11
T	-0.31	1.79	2.53	1.98		
P	0.76	0.09	0.01	0.06		
Visual analog pain score, VAS						
Control	5.9 ± 1.1	5.6 ± 1.0	5.7 ± 0.9	5.9 ± 1.0	0.29	0.83
LIPUS	6.0 ± 0.4	5.3 ± 0.5	4.6 ± 0.6	3.9 ± 0.7	33.70	<0.01
T	0.31	-0.97	-3.67	-5.91		
P	0.76	0.34	<0.01	<0.01		

Number of CPCs and cell migration

**Figure 3:**
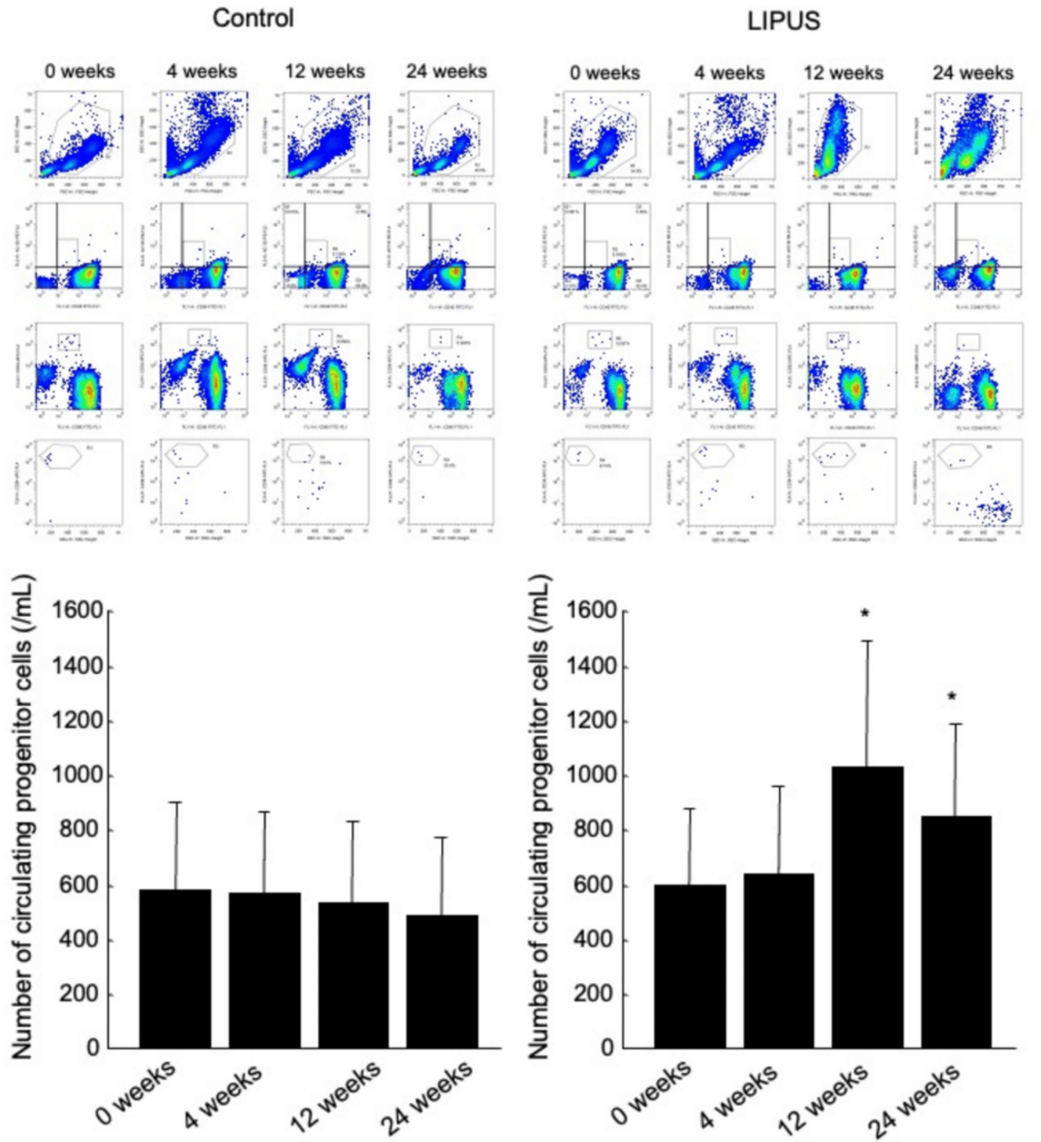
Representative measurements of the number of circulating progenitor cells by flow cytometry in patients with atherosclerotic peripheral arterial disease (PAD) in the control and the low-intensity pulsed (LIPUS) groups at the beginning of treatment (0 weeks) and after 4, 12, and 24 weeks of treatment (top). Comparison of the numbers of circulating progenitor cells in patients with atherosclerotic PAD in the control and the LIPUS groups at the beginning of treatment (0 weeks) and after 4, 12, and 24 weeks of treatment (bottom). *p<0.05 vs. 0 weeks in the same group. Abbreviations: LIPUS, low-intensity pulsed ultrasound

**Figure 4:**
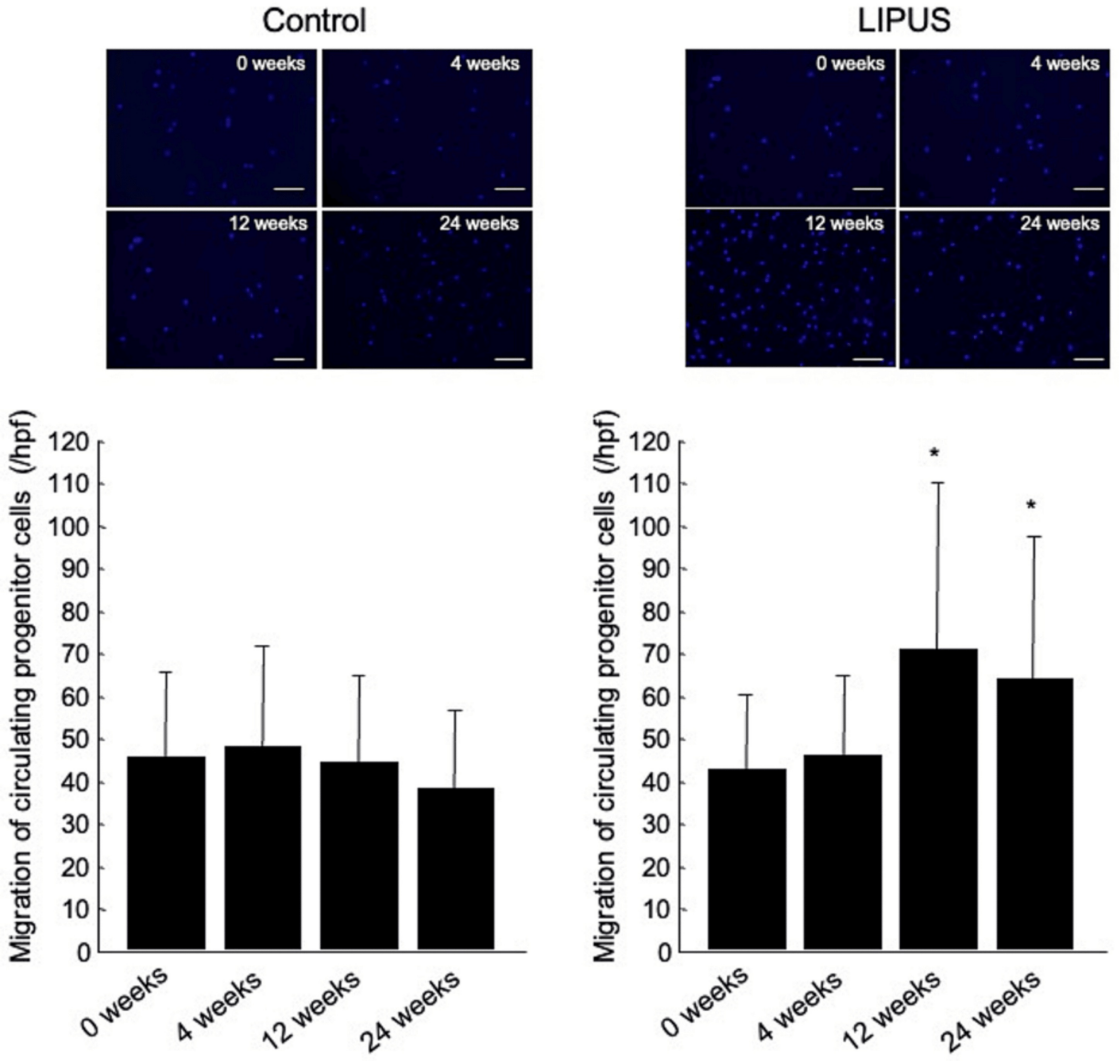
Representative measurements of the migration of circulating progenitor cells labeled with DAPI by fluorescence in patients with atherosclerotic peripheral arterial disease (PAD) in the control and the low-intensity pulsed ultrasound (LIPUS) groups at the beginning of treatment (0 weeks) and after 4, 12, and 24 weeks of treatment (top). Comparison of the migration of circulating progenitor cells in patients with atherosclerotic PAD in the control and the LIPUS groups at the beginning of treatment (0 weeks) and after 4, 12, and 24 weeks of treatment (bottom). *p<0.05 vs. 0 weeks in the same group. Abbreviations: DAPI, 4′,6-diamidino-2-phenylindole dihydrochloride; LIPUS, low-intensity pulsed ultrasound.

**Figure 5:**
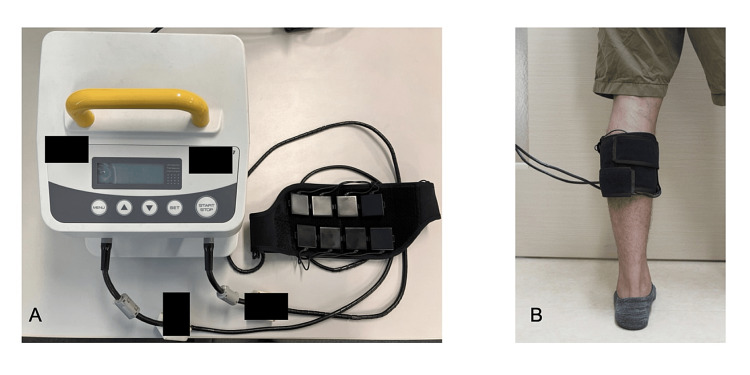
Supplementary Figure S1. (A) The low-intensity pulsed ultrasound (LIPUS) device and transducers for LIPUS irradiation. (B) Application of transducers for LIPUS irradiation in the gastrocnemius of the ischemic leg (application is as shown on a model’s leg in the picture).

**Figure 6:**
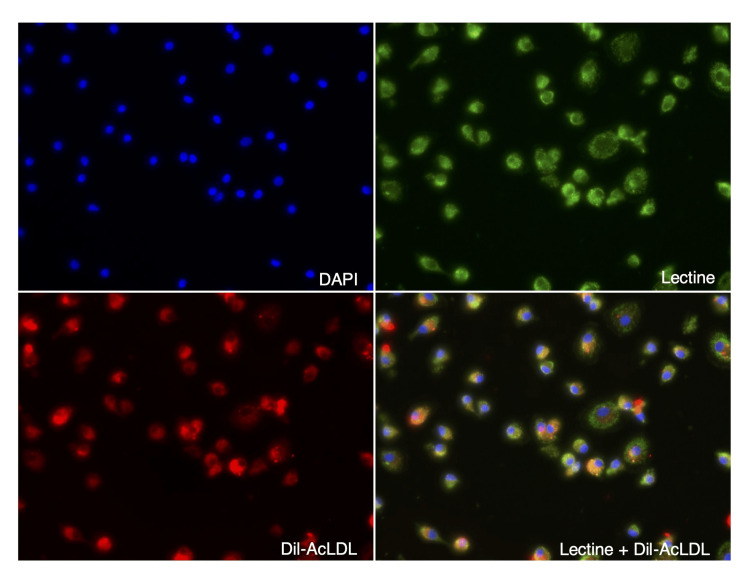
Supplementary Figure S2. Characterization of progenitor cells by immunofluoresence for lectin binding (green), 1,1’-dioctadecyl-3,3,3,3-tetramethylindocarbocyanine-labeled acetylated low-density lipoprotein (Di-AcLDL) uptake (red), and lectin/Dil-AcLDL double-positive cells (yellow) (x100).

**Figure 7:**
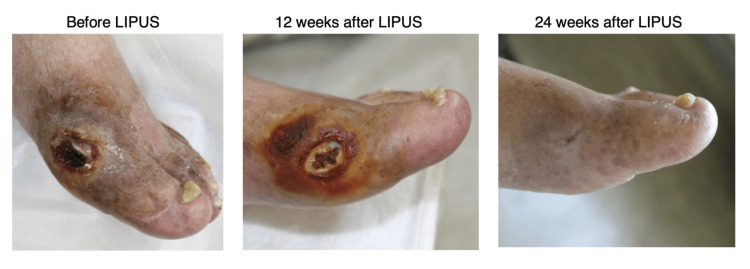
Supplementary Figure S3. Representative data on low-intensity pulsed ultrasound (LIPUS)-induced cure of ulcers in a patient with atherosclerotic peripheral arterial disease at the beginning of treatment (0 weeks), after 12 weeks of treatment, and 24 weeks of treatment.

The number of CPCs and cell migration responses to VEGF in patients with atherosclerotic PAD in the control and LIPUS groups at the 0 week of treatment (as baseline) and after 4, 12, and 24 weeks of LIPUS irradiation are shown in Figures 3-4. LIPUS irradiation for 12 weeks increased the number of CPCs from 612±280 to 1018±476/mL (p=0.01) (Figure 3) and increased to cell migration response to VEGF from 43±19 to 71±39/high-power field (p=0.01) (Figure 4). The ability of LIPUS to increase the number of CPCs and cell migration response to VEGF was maintained throughout the 24-week treatment period (852±342/mL and 64±34/high-power field, vs. baseline, p=0.03 and p=0.02, respectively). The number of CPCs and cell migration responses to VEGF after 12 and 24 weeks of treatment were similar. There was no significant difference between the number of CPCs and cell migration response to VEGF at baseline (0 week) and those at 4, 12, and 24 weeks in the control group.

Adverse effects of LIPUS

There was no report of severe adverse effects in the patients who were treated with LIPUS.

## Discussion

This is a pilot study showing the safety of LIPUS and its efficacy for symptoms in patients with atherosclerotic PAD using a randomized, double-blinded, and placebo-controlled design. There was a significant improvement in pain symptoms after LIPUS treatment in patients with PAD. Pain is one of the major clinical symptoms of PAD, and we found a 35% reduction in VAS score following LIPUS treatment, greater than the 30% threshold for clinically meaningful pain relief [19,20]. Concomitant assessments of perfusion parameters ABI, TcPO_2_, and SPP indicated that the severity of chronic limb ischemia did not differ significantly at baseline between the control and LIPUS groups. Numerical improvements in the LIPUS group suggest reduced ischemia, but the p-values did not reach statistical significance or were of borderline significance. For instance, ABI showed some improvement in the LIPUS group, but the p-value was not significant. Similarly, the change in SPP was not statistically significant at 24 weeks. However, TcPO_2_ was significantly increased in the treatment group. These parameters were examined in subjects with severe ischemic peripheral vascular disease to determine the clinical relevance of perfusion parameter outcomes. LIPUS may be a new therapeutic option for improving pain symptoms in chronic limb ischemia.

The current LIPUS protocol has been optimized and was used in the present study [10,14]. Several mechanisms have been proposed to explain how LIPUS relieves symptoms in patients with PAD. The results of in vitro and in vivo studies suggested that LIPUS promotes angiogenesis through increases in angiogenic growth factors and various cytokines, as well as effects to promote therapeutic anti-inflammatory properties [10,11,21-23]. The oscillating pressure waves induced by LIPUS were found to penetrate the skin into skeletal muscle. We have reported simulations predicting that LIPUS increases deep tissue temperature by a maximum of only 0.006°C [14], and there was no significant difference in skin temperature between the LIPUS-exposed and non-exposed legs during the study. Ultrasound also influences an increase in mechanical shear stress on the surface of endothelial cells [24]. Increasing shear stress over a period of time has been shown to alter the vascular endothelium, resulting in enhanced vascular structure and function [25]. Ultrasound may act as a mechanical stimulator of NO activation and synthesis through an increase in shear stress and an increase in intracellular Ca2+ in endothelial cells, leading to angiogenesis. However, the present study did not measure these effects directly; rather, these are hypothesized mechanisms based on previous studies. Nonetheless, LIPUS appears to induce angiogenesis and improve both perfusion and pain symptoms in patients with PAD. LIPUS is inferred to induce angiogenesis and contributes to the improvement of perfusion parameters, trends, and symptoms in patients with PAD.

Assessments of CPC numbers and migration capacities were included as these parameters are predictive of vascular regeneration outcomes. LIPUS increased the number of CPCs and the cell migration response to VEGF in patients with PAD. A higher CPC count in the presence of risk factors is associated with better vascular function among young individuals and with lower future vascular dysfunction risk [26]. A reduction in the levels of circulating cells putatively provided with vasculo-regenerative properties represents a risk factor for adverse cardiovascular outcomes and death [27,28]. LIPUS has been shown to promote the enhancement of proliferation by activating focal adhesion components, including integrin, its receptors, and Src, and the Src/Rho-associated kinase/ERK signaling pathway in human fibroblast cells [29]. It is also likely that LIPUS induces phosphorylation of ERK1/2 via focal adhesion components in endothelial cells. Toyama et al. [30] showed that LIPUS of 100 mW/cm^2^ in intensity for 240 seconds augmented the proliferation of CPCs derived from human peripheral blood mononuclear cells through enhancement of the Akt/eNOS/NO pathway in vitro. As a result of LIPUS treatment, patients with atherosclerosis in this study displayed restored generation abilities of CPCs that were derived from peripheral blood mononuclear cells, and neovascularization was found to be greater in ischemic limbs of mice in which CPCs pretreated with LIPUS had been implanted than in ischemic limbs of control mice.

PAD is one of the major complications of atherosclerosis with debilitating symptoms [1-5]. As a result of LIPUS treatment, patients with atherosclerosis in this study displayed restored generation abilities of CPCs that were derived from peripheral blood mononuclear cells. The number of and function of CPCs, including endothelial progenitor cells, play an important role in the prevalence of cardiovascular morbidity and mortality, as well as angiogenesis [26-28]. While these values were highly variable, there were significant differences between the groups. For instance, CPC numbers were significantly higher in the treatment group at 12 weeks, although numbers declined slightly by 24 weeks. Cell migration also improved but stabilized after 12 weeks. As these patients were receiving regular LIPUS intervention, the rise and plateau effects may indicate biological responses and adaptation. Alternatively, there were no significant changes in the numbers and migration capacities of CPCs among the control group. LIPUS-induced increase in the number of CPCs and LIPUS-induced enhancement of the function of CPCs may contribute not only to the improvement of symptoms but also may contribute to the reduction in the burden of cardiovascular diseases.

Current conventional treatments for revascularization focus on patients presenting with feasible conduits and fit for invasive procedures. However, many patients do not respond to current treatments, including exercise and pharmacological therapies. Treatment focus must shift to provide therapeutic effects not only on the vasculature but also on surrounding tissues. The oscillating sound pressure waves induced by LIPUS have been shown to influence angiogenesis, myogenesis, osteogenesis, and wound healing. Atherosclerotic and non-atherosclerotic PADs, such as Buerger disease and other conditions, may benefit from LIPUS treatment. Indeed, Mohamad Yusoff et al. [14] reported that LIPUS irradiation was safe and improved symptoms in patients with Buerger disease. In the present study, LIPUS was found to benefit severe PAD patients with atherosclerosis. It is expected that the use of LIPUS will increase as an option for PAD patients of widely varying limb ischemia severity, either as an adjunct therapy to conventional strategies or as a standalone option.

Study limitations

As a first point, in this study, the number of subjects was relatively small. Nonetheless, after LIPUS irradiation, improvement of symptoms was observed in treated patients. Numerical improvements in perfusion parameters among patients receiving LIPUS suggest reduced ischemia, but the p-values did not reach statistical significance or were of borderline significance. Thus, studies with larger sample sizes are necessary to confirm these responses. Second, the optimal ultrasound treatment method for improving symptoms of limb ischemia in humans remains unclear. Prior studies have reported that LIPUS increases shear stress, NO synthesis, and intracellular Ca2+ levels, but the present study did not measure these effects directly. Moreover, these are hypothesized mechanisms based on previous studies rather than proven mechanisms in the present study. A better understanding of the role of ultrasound in angiogenesis in humans can be gained by establishing an optimal LIPUS treatment and elucidation of the underlying mechanisms. Third, in this study, no other migratory cell population was included as a positive control for validation. Fourth, although no severe adverse effects were detected in the present study, further studies are necessary to examine adverse effects and outcomes, including both cardiovascular outcomes, limb salvage, MACE, mortality, functional mobility improvements, and the development of malignancies. There is thus a further need for prospective randomized controlled trials with linger follow-up, as well as larger-scale multicenter trials including specific patient subgroups, such as equal numbers of males and females, diabetic PAD patients, patients with or without arterial calcifications, and patients receiving revascularization or other therapeutic strategies (e.g., exercise and pharmacological treatments).

## Conclusions

In conclusion, LIPUS is a safe, noninvasive treatment option to improve symptoms in patients with atherosclerotic PAD. Patients with chronic and critical limb-threatening ischemia are in need of an innovative and safe therapeutic option to relieve their ischemic limb symptoms and to effectively facilitate improvement in their quality of life. The observed effects of LIPUS on CPC numbers and migration capacities suggest that patients with mild, moderate, or severe chronic limb ischemia can benefit from this treatment strategy. Atherosclerotic and non-atherosclerotic PADs, such as Buerger disease and other conditions, may also benefit from LIPUS treatment. It is expected that the use of LIPUS will increase as an option for PAD patients, either as an adjunct therapy to conventional strategies or as a standalone option. Further studies are needed to examine long-term effects and overall outcomes.
